# Combining scores from different patient reported outcome measures in meta-analyses: when is it justified?

**DOI:** 10.1186/1477-7525-4-94

**Published:** 2006-12-07

**Authors:** Milo A Puhan, Irene Soesilo, Gordon H Guyatt, Holger J Schünemann

**Affiliations:** 1 Horten Centre, University of Zurich, Switzerland; 2 Department of Medicine, State University of New York at Buffalo, New York, USA; 3 Department of Clinical Epidemiology and Biostatistics, McMaster University, Hamilton, Ontario, Canada; 4 Clinical Research Development and Information Translation (INFORMA) Unit, Department of Epidemiology, Italian National Cancer Institute Regina Elena, Rome, Italy

## Abstract

**Background:**

Combining outcomes and the use of standardized effect measures such as effect size and standardized response mean across instruments allows more comprehensive meta-analyses and should avoid selection bias. However, such analysis ideally requires that the instruments correlate strongly and that the underlying assumption of similar responsiveness is fulfilled. The aim of the study was to assess the correlation between two widely used health-related quality of life instruments for patients with chronic obstructive pulmonary disease and to compare the instruments' responsiveness on a study level.

**Methods:**

We systematically identified all longitudinal studies that used both the Chronic Respiratory Questionnaire (CRQ) and the St. George's Respiratory Questionnaire (SGRQ) through electronic searches of MEDLINE, EMBASE, CENTRAL and PubMed. We assessed the correlation between CRQ (scale 1 – 7) and SGRQ (scale 1 – 100) change scores and compared responsiveness of the two instruments by comparing standardized response means (change scores divided by their standard deviation).

**Results:**

We identified 15 studies with 23 patient groups. CRQ change scores ranged from -0.19 to 1.87 (median 0.35, IQR 0.14–0.68) and from -16.00 to 3.00 (median -3.00, IQR -4.73–0.25) for SGRQ change scores. The correlation between CRQ and SGRQ change scores was 0.88. Standardized response means of the CRQ (median 0.51, IQR 0.19–0.98) were significantly higher (p < 0.001) than for the SGRQ (median 0.26, IQR -0.03–0.40).

**Conclusion:**

Investigators should be cautious about pooling the results from different instruments in meta-analysis even if they appear to measure similar constructs. Despite high correlation in changes scores, responsiveness of instruments may differ substantially and could lead to important between-study heterogeneity and biased meta-analyses.

## Background

Systematic reviews and meta-analyses should include all available evidence to avoid selection bias and to increase the power of analyses of primary effects and effect modification by differences in patients and interventions. In meta-analysis of patient reported outcome (PRO) measures, effects are, however, often measured with different instruments. For example, meta-analyses of respiratory rehabilitation in chronic obstructive pulmonary disease (COPD) are typically either based on studies using the Chronic Respiratory Questionnaire (CRQ)[1] or the St George's Respiratory Questionnaire (SGRQ) [2-4].

Investigators often deal with this challenge by standardizing scores from different instruments and combining them as unit-free scores – effect sizes or standardized response means (SRMs)[2,5,6] However, critics have noted that, because standard deviations (SD) may vary substantially from study to study, treatment effects that are homogeneous when expressed in their original unit can become heterogeneous when expressed as (SRM) [7]. An alternative to standardisation is to directly transform PRO scores. For instance, investigators could transform CRQ into SGRQ scores or vice versa using transformation coefficients from regression analyses [8].

For either method of combining scores, two important premises must ideally be met. First, the scores must correlate strongly indicating that the instruments measure constructs that are similar enough to be combined. Second, the responsiveness – the instrument's ability to detect important changes even if those changes are small – should be similar. If instruments express different magnitude of change for identical underlying effects, less responsive instruments will underestimate treatment effects, and meta-analyses will manifest heterogeneity that might falsely be attributed to variability in patient or interventions or effects of the interventions.

While investigators found moderate to strong correlations between the CRQ and SGRQ on an individual patient level [9-12] suggesting that they provide similar information, it is unknown whether a strong relationship between CRQ and SGRQ change scores exists on a study level. The objective of this study was to assess the correlation of CRQ and SGRQ change scores as well as their responsiveness on a study level and to evaluate the implications for combining scores in meta-analyses.

## Methods

We conducted a systematic literature search to identify all longitudinal studies that used both the CRQ and SGRQ.

### Search strategy

We began the literature search by identifying all studies that used the CRQ using the keywords "chronic respiratory questionnaire", "chronic respiratory disease questionnaire", "CRQ" and "CRDQ" for electronic database searches in MEDLINE (Ovid version, New York, New York, from inception to November 2004), EMBASE (DataStar version, Cary, North Carolina from inception to November 2004) and the Cochrane Central Register of Controlled Trials (Oxford, United Kingdom, 2004, Issue 4). We also used the related articles feature in PubMed (National Library of Medicine, Washington, Maryland) for included articles to search for additional papers. In addition, we hand-searched the bibliographies of included primary studies and our own files.

### Study selection criteria

#### Study design

Eligible studies included both randomized controlled trials and uncontrolled studies with a baseline measurement and at least one follow-up measurement of the CRQ and SGRQ.

#### Participants

We included studies if more than 90% of study participants had COPD defined by chronic airflow obstruction (FEV_1 _less than 80% predicted) and little reversibility of airflow obstruction (reversibility of FEV1 in % predicted in response to inhaled β-agonists below 20%).

#### Interventions

Any intervention, usual care, placebo or time (natural history).

#### Outcome measures

Studies had to include both the CRQ and SGRQ.

#### Study selection

**Figure 1:**
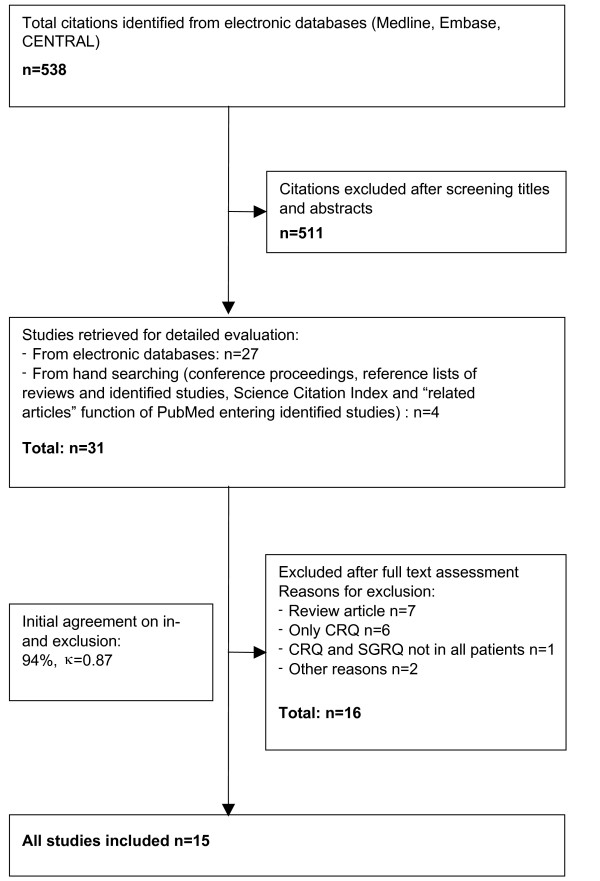
Study flow from electronic database searches to final inclusion of eligible studies.

Two members of the study team independently scrutinized the titles and abstracts of all identified citations (see Figure 1). We obtained the full text of any article that was deemed potentially eligible by one of the reviewers. The two reviewers then evaluated the full text of all retrieved papers and evaluated their eligibility, resolving disagreement by consensus.

#### Data extraction

Information extracted included details about patients, interventions, length of follow-up, study design, mean change scores (difference between follow-up scores and baseline) for the CRQ total and SGRQ total scores, and SD of CRQ total and SGRQ total baseline and change scores. If the SD was not available from reports we used the median SD from studies that reported the SD. For studies, for which a CRQ total score was not provided in the article, we calculated the mean of the four domain change scores. Because one cannot calculate SGRQ total scores on the basis of domain scores, we asked authors of articles reporting only SGRQ domain scores to provide total scores.

#### Quality assessment

We evaluated two aspects of study quality. First, we assessed whether the order of administration of the CRQ and SGRQ was randomised and second, whether investigators used validated versions of the CRQ and SGRQ. We considered questionnaires to be validated if the investigators referred to a reference for the validation process in the respective language.

### Statistical analysis

We performed all analyses on a study level. We first calculated median CRQ and SGRQ scores together with their interquartile ranges (25^th ^to 75^th ^percentile). We then assessed the relationship between CRQ and SGRQ change scores using scatter plots and Spearman rank correlation coefficients. Our criterion for a strong correlation, 0.7 or more, exceeded that of previous studies with individual patient data (0.5) because the use of mean change scores is likely to increase correlation coefficients by lowering denominators of the correlation coefficients.

To compare the responsiveness of the CRQ and SGRQ we calculated SRMs by dividing change scores by the SD of change cores and multiplying the resulting SGRQ SRM by -1 to adjust for the fact that negative scores indicate improvement on the SGRQ [13]. We then conducted a Wilcoxon signed-rank test. We performed all statistical analyses using SPSS for Windows version 12.0.1 (SPSS Inc, Chicago, Ill).

## Results

The electronic database search yielded 538 citations of which 27 articles were potentially eligible (Figure 1); hand searching added another 4 articles. Full text review of these 31 articles demonstrated that 15 studies fulfilled our inclusion criteria. Most of the 16 excluded studies were review articles or included only the CRQ. Agreement on inclusion and exclusion was excellent (agreement in 94% of all decisions, chance-corrected kappa = 0.87).

Table 1 describes the 15 included studies that reported on 22 patient groups. In 11 groups, patients followed a respiratory rehabilitation program; 9 groups were cohorts without specific interventions or controls in randomized controlled trials receiving usual care, patient education or placebo and in two groups, patients received inhaled bronchodilators. Sample size ranged from 21 to 183 and duration of follow-up from 4 to 52 weeks. In all studies, investigators used validated versions of the CRQ and SGRQ and in four studies they randomized the order of administration of the CRQ and SGRQ. The results published by Bestall [14] and Wedzicha [15] were based on the same randomized controlled trial. We only included the last available data for each patient group, i.e. the 52 weeks follow up data for patients with moderate to severe COPD and the 8-week follow-up data for patients with severe COPD. For one study, mean CRQ and SGRQ total change scores were not available [16].

**Table 1:** Characteristics of included studies

Study groups	Number of patients	Intervention	Wks of follow-up	Study design	Mean baseline scores (SD)		Mean change scores (SD)		Order of questionnaires randomized	Use of validated questionnaires
									
					**CRQ**	**SGRQ**	**CRQ**	**SGRQ**		
Barr [18]	102	Respiratory rehabilitation (RP)	4	Prospective follow-up study	3.89 (0.89)	55.8 (17.9)	0.71 (0.75)	-3.00 (12.20)	No	Yes
Bestall [14]	23	RP	52	RCT	4.00 (0.90)	51.0 (13.7)	0.35 (0.85)	-3.00 (12.20)	No	Yes
Bestall [14]	21	Patient education	52	RCT	4.28 (0.98)	51.9 (13.9)	-0.05 (0.58)	-1.00 (9.35)	No	Yes
Bourbeau [22]	65	RP	2	Prospective follow-up study	n.a.	n.a.	0.74 (0.74)	-7.20 (12.30)	Yes	Yes
Bourbeau [22]	27	Usual care	2	Prospective follow-up study	n.a.	n.a.	0.35 (0.31)	-2.40 (5.57)	Yes	Yes
Connor [16]	170	RP	52	Prospective follow-up study	n.a.	51.6 (15.8)	n.a.	n.a.	No	Yes
Desikan [23]	40	Usual care	12	Prospective follow-up study	3.75 (0.95)	50.2 (18.5)	0.33 (0.86)	-2.51 (9.71)	Yes	Yes
de Torres [19]	37	RP	8	Prospective follow-up study	4.13 (0.95)	52.0 (13.0)	0.56 (1.20)	-4.00 (13.40)	No	Yes
Griffiths [20]	37	RP	52	RCT	3.59 (0.95)	64.9 (12.7)	0.25 (0.80)	-3.40 10.86)	No	Yes
Griffiths [20]	33	Usual care	52	RCT	3.53 (0.95)	68.3 (13.3)	-0.08 (0.80)	0.70 (10.86)	No	Yes
Hajiro [24]	165	Usual care	12	Prospective follow-up study	5.31 (0.97)	40.3 (20.1)	0.65 (0.83)	-6.00 (18.50)	No	Yes
Harper [25]	156	Usual care	26	Prospective follow-up study	3.69 (0.95)	65.4 (15.9)	0.07 (1.00)	0.81 (9.95)	No	Yes
Man [17]	21	RP	18	RCT	2.64 (0.95)	65.4 (13.5)	1.87 (0.53)	-16.10 (5.95)	No	Yes
Man [17]	21	Usual care	16	RCT	3.11 (0.95)	69.6 (13.6)	0.49 (0.26)	-3.40 (8.50)	No	Yes
Rutten [9]	47	Salmeterol + Ipratropium	12	RCT	4.44 (0.81)	48.3 (13.9)	0.20 (0.60)	-2.40 (11.00)	No	Yes
Rutten [9]	47	Salmeterol + Placebo	12	RCT	4.58 (1.03)	50.5 (16.6)	-0.19 (0.80)	-0.01 (7.10)	No	Yes
Rutten [9]	50	Placebo	12	RCT	4.22 (1.05)	50.2 (15.8)	0.03 (0.70)	0.50 (9.20)	No	Yes
Singh [12]	97	RP	7	Prospective follow-up study	3.66 (0.92)	58.8 (15.2)	0.72 (1.00)	-4.02 (12.98)	No	Yes
Schunemann [8]	84	RP	12	Prospective follow-up study	4.01 (0.95)	52.8 (13.9)	1.14 (0.90)	-8.10 (20.40)	Yes	Yes
Schunemann [10]	183	RP	12	Prospective follow-up study	4.57 (0.95)	50.6 (13.9)	0.55 (0.89)	-5.43 (11.18)	Yes	Yes
Wedzicha [15]	26	RP on	8	RCT	3.76 (0.90)	56.7 (14.0)	0.20 (0.39)	3.00 (10.40)	No	Yes
Wedzicha [15]	28	Patient education	8	RCT	3.82 (1.20)	59.1 (13.0)	0.20 (0.32)	2.00 (8.10)	No	Yes

RCT: Randomized controlled trial; n.a.: not available

Mean total scores at baseline ranged from 2.64 to 5.31 for the CRQ and from 40.3 to 69.6 for the SGRQ. The correlation coefficient for total scores at baseline was -0.86 (95% CI 0.62–1.00).

**Figure 2:**
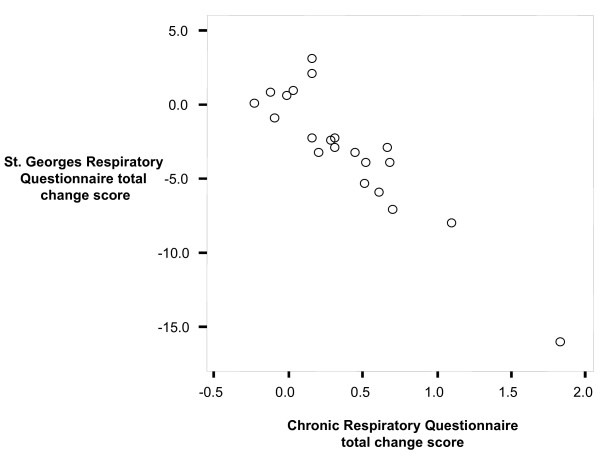
Scatter plots of CRQ and SGRQ change scores from 21 groups where patients completed both the CRQ and SGRQ.

CRQ change scores ranged from -0.19 to 1.87 (median 0.35, IQR 0.14–0.68) and for the SGRQ from -16.10 to 3.00 (median -3.00, IQR -4.73–0.25). Figure 2 shows the strong correlation between CRQ and SGRQ change scores with a correlation coefficient of 0.88. One study [17] showed substantially larger effects than the others on both instruments and could have led to this strong correlation. However, a sensitivity analysis excluding the study by Man et al [17] showed that it had little influence on the correlation coefficient (r = 0.86).

**Figure 3:**
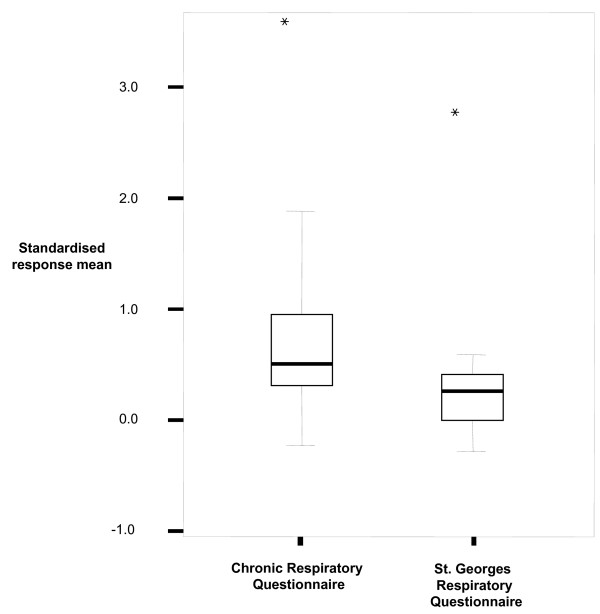
Box plots showing the distributions of standardised response means for the CRQ and SGRQ. Boxes display the interquartile range and the median while the whiskers show 95% of the data. Asterisks indicate an outlier study group [17] that showed much larger change scores that the other studies.

Figure 3 shows the SRMs for the CRQ and SGRQ. SRMs ranged from -0.24 to 3.53 for the CRQ (median 0.51, IQR 0.19–0.98) and were significantly higher (p < 0.001) compared to standardised response means of the SGRQ (range from -0.29 to 2.71, median 0.26, IQR -0.03–0.40).

## Discussion

We observed that high correlation between PRO measure change scores does not necessarily imply similar responsiveness. In our example of the CRQ and SGRQ we showed a strong correlation between total change scores on a study level, but the CRQ was substantially more responsive than the SGRQ. This finding indicates that these two measures provide very similar information and could justify the use of pooled estimates in meta-analyses on a conceptual, theoretical level. However, studies using the less responsive measure are likely to underestimate treatment effects and to introduce heterogeneity in study results.

Strengths of this study include the systematic review approach to identifying longitudinal studies using both the CRQ and SGRQ. A limitation of our approach is the lack of individual patient data to explore the association between CRQ and SGRQ change scores in greater detail.

Earlier studies indicated that the responsiveness of the CRQ is superior to the SGRQ when applied to the same patients [12,13]. Our results extend these findings beyond the previous samples and demonstrate its generalizability. The phenomenon will not only lead to underestimates of effect in studies using the SGRQ, but could lead investigators who are unaware of different responsiveness to spuriously attribute variability to differences in patients, interventions, intervention effects or methodological quality.

The rehabilitation studies included in this systematic review indicate the extent of underestimation by the SGRQ. In the nine studies with patients following respiratory rehabilitation[8,10,12,14,15,17-20], the median SGRQ change was 4.0 points or, expressed as SRM 0.31. The corresponding SRM based on the CRQ change scores was 0.62. If the SRM of 0.62 was expressed as SGRQ change scores the corresponding change would be 8.0 points. The difference of 4 points between changes measured by the CRQ and SGRQ is substantial and equivalent to the minimal important difference of the SGRQ or to the shift from a minimal to a moderate difference [8].

A conservative solution to the problem with meta-analysis that we raised is to restrict analyses to the most responsive available instrument. Investigators could develop alternatives allowing for combining trials with different instruments. Preferable alternatives to this conservative approach would include testing for an association between effect size and the outcome measure and, if there is no association within the individual meta-analysis, using all studies irrespective of outcome measure. Alternatively, investigators could introduce instrument as a variable in meta-regression models. Finally, if there is a strong linear relationship between instruments (as in this case), one could transform the scores of one instrument into those of another instrument. For example, SGRQ scores could be transformed into CRQ scores using the equation of a linear regression model where SGRQ was used to predict CRQ scores[8]

In theory, reasons for the superior responsiveness of the CRQ could include statistical reasons, differences in the aspects of HRQL measured by the CRQ and SGRQ, and the way these questionnaires are administered. A statistical reason why the CRQ is more responsive is the lower variability of CRQ scores leading to smaller noise terms. The domains of CRQ and SGRQ do not measure identical aspects of HRQL even though their total scores correlate highly. The domains of SGRQ focus on impairment from respiratory symptoms while the CRQ also addresses impairment from extra-pulmonary manifestations of COPD such as fatigue or depressive symptoms. Thereby, it is possible that the CRQ captures, by its broader approach, improvements of pulmonary and extra-pulmonary manifestation better than the SGRQ. However, the corresponding domains on the two questionnaires (e.g. symptoms and impact on the SGRQ compared with dyspnea and physical functioning) generally show greater responsiveness for the CRQ indicating that this may not be the explanation. Finally, the administration format may also influence responsiveness. In two randomised trials where we compared the interviewer- and self-administered CRQ[10,11] we found that the self-administered CRQ tends to be more responsive than the interviewer-administered CRQ. This was mainly due to lower baseline scores with the self-administered format. Patients may be more willing to express the severity of impairment in the absence of an interviewer. Thus self-administration might enhance responsiveness compared with interviewer-administration. The SGRQ is a self-administered questionnaire and the CRQ required, until recently and as it was the case in the studies of this systematic review, an interviewer. If self-administration is associated with greater responsiveness the analyses presented in this article may even underestimate differences in responsiveness between the CRQ and the SGRQ.

## Conclusion

The presence of a strong relationship of two different instruments alone does not allow combining them in meta-analysis. There should be similar responsiveness, otherwise pooled estimates may become biased and substantial heterogeneity can arise. At present, investigators should remain cautious about combining results from trials that use different instruments without careful exploration of possible heterogeneity of the results.

## Competing interests

MAP and IS declare that they have no competing interests. HJS and GHG declare that they have no competing interests with the present analysis but they are the principal authors of the CRQ.

## Authors' contributions

MP, HJS and GHG participated in the design of the study. MP and IS collected the data. MP, HJS and GHG carried the statistical analysis and drafted the manuscript. All authors read and approved the final manuscript.

## Author disclosure

The CRQ is copyrighted by McMaster University, Hamilton, Canada; Principal Authors Dr. Gordon Guyatt and Dr. Holger Schünemann. Use of the CRQ requires permission by McMaster University and the authors.
